# Persistent Neutropenia and Atopy in an Adolescent: A Subtle Presentation of Phosphoglucomutase 3 Deficiency

**DOI:** 10.7759/cureus.86531

**Published:** 2025-06-22

**Authors:** Madalena Fonseca, Francisco Abrantes, Sara Pinho, Isabel Esteves, Catarina Salgado, Carolina Gonçalves, Anabela Ferrão

**Affiliations:** 1 Pediatrics, Hospital de Santa Maria, Unidade Local de Saúde de Santa Maria, Lisbon, PRT; 2 Pediatrics and Genetics, Hospital de Santa Maria, Unidade Local de Saúde de Santa Maria, Lisbon, PRT; 3 Pediatrics, Infectious Diseases, and Immunodeficiencies, Hospital de Santa Maria, Unidade Local de Saúde de Santa Maria, Lisbon, PRT; 4 Pediatrics, Unidade Local de Saúde do Alentejo Central, Evóra, PRT; 5 Pediatrics and Hematology, Hospital de Santa Maria, Unidade Local de Saúde de Santa Maria, Lisbon, PRT

**Keywords:** chronic neutropenia, congenital disorder of glycosylation, eczema, inborn errors of immunity, pgm3 deficiency

## Abstract

Phosphoglucomutase 3 (PGM3) deficiency (OMIM (Online Mendelian Inheritance in Man) #615816) is a rare autosomal recessive congenital disorder of glycosylation that disrupts multiple glycosylation pathways, with few cases reported in the literature. It leads to a broad clinical spectrum ranging from hyper-IgE syndrome (HIES)-like features to severe combined immunodeficiency (SCID). We report a case of a 17-year-old female of Brazilian origin, referred to our center in Portugal for investigation of persistent neutropenia. Her medical history included recurrent infections in early childhood, severe eczema, and autism spectrum disorder. She exhibited persistent neutropenia and T-cell lymphopenia, with elevated IgE levels. Genetic analysis using a next-generation sequencing panel for primary immunodeficiencies identified compound heterozygous likely pathogenic variants in PGM3: a missense variant (c.1475C>T, p.(Thr492Ile)) and a complete gene deletion in the other allele, confirming the diagnosis of PGM3 deficiency. Chronic neutropenia was the main finding that prompted the genetic investigation. Although it is not a defining feature of PGM3 deficiency, it has been reported in nearly half of the cases. In this patient, the clinical presentation has been comparatively milder than the severe phenotypes described in the literature, which highlights the phenotypic variability of this condition and the need for clinical suspicion, even when classical features are absent. The genetic diagnosis has important implications for clinical follow-up and enables appropriate genetic counseling. This case illustrates the clinical variability of PGM3 deficiency and reinforces the role of genetic testing in clarifying the diagnosis, guiding management, and informing long-term follow-up in rare inborn errors of immunity.

## Introduction

Phosphoglucomutase 3 (PGM3) deficiency (OMIM (Online Mendelian Inheritance in Man) #615816) is a rare autosomal recessive congenital disorder of glycosylation that affects the synthesis of UDP-GlcNAc, a key substrate in multiple glycosylation pathways [1]. Glycosylation is a fundamental post-translational modification process, involving approximately 1-2% of the human genome [1]. This mechanism consists of the covalent attachment of carbohydrate chains (glycans) to proteins or lipids and plays a crucial role in numerous physiological functions, including immune system regulation [1,2].

Human PGM3 is an essential enzyme in this process, catalyzing the conversion of N-acetylglucosamine (GlcNAc)-6-phosphate to GlcNAc-1-phosphate, a key step in the biosynthesis of uridine diphosphate (UDP)-GlcNAc [3-6]. UDP-GlcNAc serves as a vital substrate in several glycosylation pathways, including N-linked and O-linked glycosylation, and is also required for the synthesis of proteoglycans and glycosylphosphatidylinositol-anchored proteins [4]. These glycosylated structures are found across a wide range of cellular locations, including the cell membrane, plasma, extracellular matrix, and intracellular compartments [4]. Additionally, UDP-GlcNAc is involved in the cytosolic and nuclear addition of O-GlcNAc residues, where it contributes to intracellular signaling pathways [4].

In 2014, three independent research groups identified autosomal recessive hypomorphic variants in the PGM3 gene across nine unrelated families [4,6-8]. Affected individuals exhibited clinical features consistent with either hyper-IgE syndrome (HIES) or combined immunodeficiency (CID) [4,6,8]. 

## Case presentation

We report the case of a 17-year-old female, born in São Paulo, Brazil, who moved to Portugal at the age of 16. She was referred to our tertiary pediatric center for evaluation of persistent neutropenia of unknown etiology.

The patient is the first and only child of non-consanguineous parents, both born in Brazil. Her mother is healthy, and her father has type 2 diabetes mellitus. She has a healthy paternal half-sister. There is no known family history of inborn errors of immunity, fetal loss, or early childhood death.

Her prenatal and perinatal history were unremarkable. She was born at term via spontaneous vaginal delivery with an appropriate birth weight. The neonatal period was uneventful, and the umbilical cord detached on the fifth day of life.

At 20 months of age, she began experiencing recurrent infections, including acute otitis media, stomatitis, and recurrent pneumonia, needing multiple courses of outpatient antibiotics. At the age of two, she required a 10-month hospitalization for severe pneumonia. Interestingly, she remained infection-free between the ages of four and six.

Since the age of six, following a Parvovirus B19 infection, she developed persistent neutropenia, with absolute neutrophil counts ranging from 100 to 1320/µL. Initial treatment with granulocyte-colony stimulating factor yielded only a partial response. A bone marrow biopsy revealed hypocellularity with a predominance of immature cells and mild dyserythropoiesis.

Concurrently, she developed diffuse eczema, managed with topical corticosteroids. A skin biopsy performed at the age of eight confirmed chronic eczematous dermatitis. 

From a neurodevelopmental perspective, the patient reached normal developmental milestones up to age five. From the age of six, she exhibited socialization difficulties that were later diagnosed as autism spectrum disorder, level 1. She also has a learning disability and is currently attending school with an adapted curriculum.

At the age of 16, she moved to Portugal and was referred to our center for further evaluation. On examination, she appeared well-nourished with no facial or limb dysmorphisms. Eczematous lesions were present in flexural areas, but no other abnormalities were noted.

**Figure 1:**
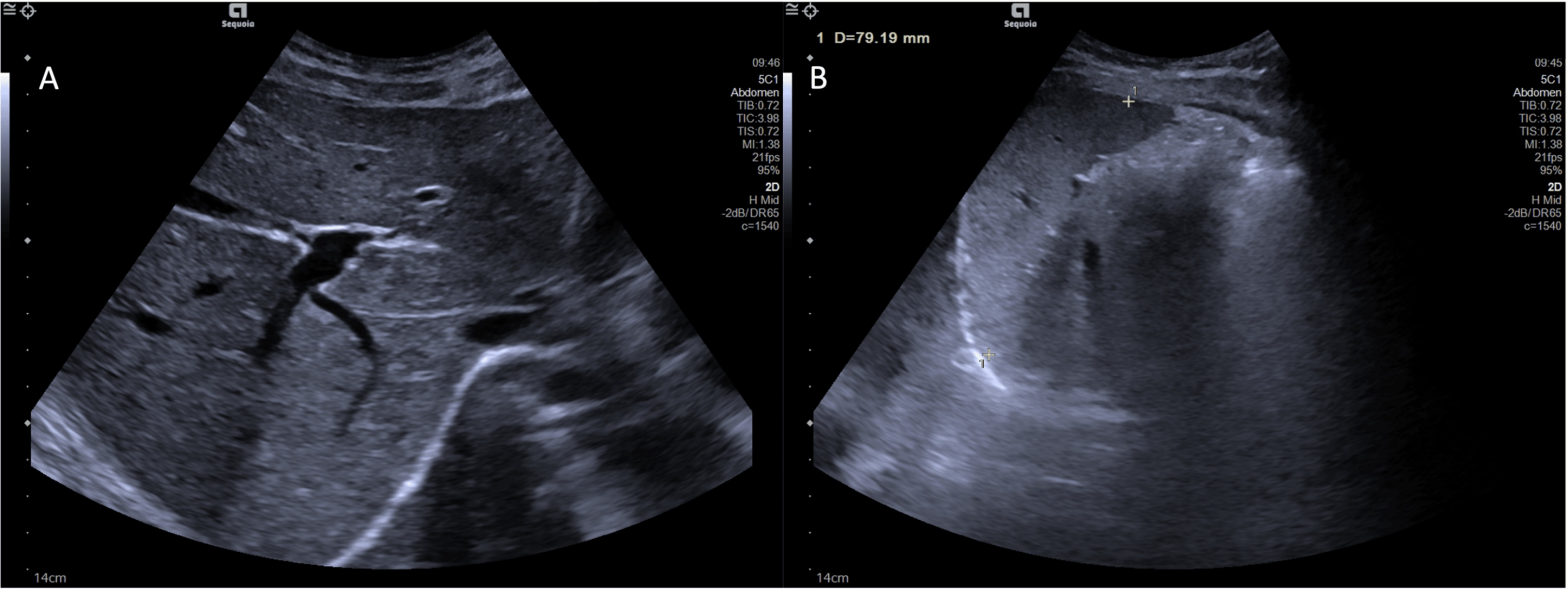
Abdominal ultrasound A) Liver of normal size, with regular contours and homogeneous echotexture. Bile ducts are not dilated, and the portal vein and hepatic veins are of normal caliber. B) Spleen of normal size (79.1 mm) and morphology. Other findings: Gallbladder with thin walls and no signs of gallstones. The pancreas has normal size and morphology. No adenopathy or gross abnormal abdominopelvic masses were observed. No intraperitoneal fluid is present.

Laboratory investigations revealed persistent neutropenia (650-1320/µL) and T-cell lymphopenia, without anemia or thrombocytopenia. IgE levels were markedly elevated (1907 U/mL). Abdominal ultrasound was unremarkable (Figure 1). Additional laboratory results are summarized in Table 1.

**Table 1:** Immunological, hematological, autoimmune, and infectious laboratory investigations performed at our center dsDNA, double-stranded deoxyribonucleic acid antibodies; ANAs, antinuclear antibodies; HIV 1/2, human immunodeficiency virus types 1 and 2; AgHBs, hepatitis B surface antigen; HCV, hepatitis C virus; CMV, cytomegalovirus; VZV, varicella-zoster virus; VCA, viral capsid antigen

Category	Test	Results		Normal range
Hematology	Total leukocytes	2.40x10^9^/L		4.0-11.0x10^9^/L
	Lymphocytes	0.97x10^9^/L (40%)		1.0-4.8x10^9^/L
	Neutrophils	0.75x10^9^/L (31%)		1.9-7.5x10^9^/L
	Eosinophils	0.38x10^9^/L (15.9%)		0.0-0.5x10^9^/L
	Hemoglobin	12.2 g/dL		12.0-15.3 g/dL
	Platelets	223x10^9^/L		150-450x10^9^/L
Immunology	Lymphocyte subpopulations	CD3+:	72.7%	60-85%
			701/uL	1000-2200/uL
		CD4+:	38.3%	29-59%
			369/uL	500-1600/uL
		CD8+:	30.6%	19-48%
			295/uL	200-800/uL
	Immunoglobulins	IgG levels: 896 mg/dL		658-1534 mg/dL
		IgA levels: 275 mg/dL		53-287 mg/dL
		IgM levels: 69 mg/dL		48-186 mg/dL
		IgE levels: 1907 U/mL		<100 U/mL
	Complement system	C3 level: 106 mg/dL		83-152 mg/dL
		C4 level: 17 mg/dL		13-37 mg/dL
		CH50 level: 58.1 U/mL		41-95 U/mL
Autoimmunity	Anti-dsDNA	Negative		
	ANAs	Negative		
	Sedimentation speed	4 mm/h		<12 mm/h
Infectious serologies	Anti-HIV 1/2	Negative		
	AgHBs	Negative		
	Anti-HCV	Negative		
	CMV	Immune (IgG+; IgM-)		
	Parvovirus	Non-immune (IgG-; IgM-)		
	Epstein-Barr virus	Immune (anti-VCA IgG+; IgM-)		
	VZV	Immune (IgG+; IgM-)		
	Measles	Non-immune (IgG-; IgM-)		

Genetic analysis using next-generation sequencing (NGS)-targeted multigene panel for primary immunodeficiencies identified two likely pathogenic variants in compound heterozygosity in the PGM3 gene: a known missense variant, c.1475C>T p.(Thr492Ile), and a complete gene deletion resulting from a 167 kb deletion on chromosome 6q. These findings confirmed a molecular diagnosis of PGM3 deficiency. The appendices include the genes targeted in the NGS panel performed.

The patient remains under regular follow-up in immunology, hematology, and dermatology clinics. She continues to exhibit persistent neutropenia and lymphopenia, with only mild infections since the age of six. To date, she has not required antibiotic prophylaxis or immunoglobulin replacement therapy and has had no further hospitalizations or significant infections.

## Discussion

PGM3 deficiency is caused by either homozygous or compound heterozygous hypomorphic pathogenic or likely pathogenic variants in the PGM3 gene, which result in a partially functional enzyme rather than a complete loss of function. Complete loss of function is presumed to be embryonically lethal, given the essential nature of the gene [5,7].

These abnormalities likely disrupt multiple pathways involved in leukocyte development and maturation. A consistent immunological feature among patients with PGM3 deficiency is the quantitative loss of circulating CD4+ T lymphocytes and a reduction in mitogen-induced proliferative capacity [7]. Although the precise role of PGM3 in CD4+ T cell development and homeostasis remains unclear, functional studies suggest that impaired T-cell receptor (TCR)-dependent proliferation, along with defects in glycolysis and mitochondrial respiration, may contribute to this phenotype [7]. Glycosylation is also required for effective TCR and CD28 co-stimulation; thus, its disruption compromises naïve T cell activation and appears to alter CD4+ T cell subset polarization, favoring Th2 differentiation while reducing the development of regulatory T cells and Th17 cells, ultimately leading to elevated serum IgE levels and eosinophilia [7].

Since its initial description in 2014, a total of 44 cases have been reported in the literature [7]. A recent comprehensive review published in 2024 by Yan et al. categorized the immunological and clinical phenotypes associated with PGM3 deficiency into three main groups: HIES-like phenotype (n=26), CID (n=7), and SCID-like presentation with marked lymphopenia (n=11) [7]. These immunological profiles are frequently accompanied by syndromic manifestations, including skeletal abnormalities and developmental delays [3,7]. The median age at symptom onset was approximately one year old [7].

Recurrent infections were reported in the majority of the patients, particularly affecting the skin, ears, respiratory tract, and gastrointestinal system [7]. The most commonly isolated pathogens included Staphylococcus aureus, Pseudomonas aeruginosa, Proteus mirabilis, Candida species, and various viruses [3,7,9].

Atopy, including eczema, asthma, and multiple allergies, is also a predominant feature, with severe eczematous dermatitis documented in 37/44 patients, and often emerging during early infancy or childhood [7].

Neurological manifestations, mainly cognitive delay, are frequent and assist in distinguishing this syndrome from other HIES, namely DOCK8 immunodeficiency syndrome or autosomal dominant HIES due to STAT3 deficiency [5,7,9].

The clinical presentation of our patient closely aligns with the features reported in the literature, as shown in Table 2. She experienced recurrent sino-pulmonary infections beginning in early childhood, including one episode of severe pneumonia requiring prolonged hospitalization, with subsequent infections managed in an outpatient setting. She also developed severe atopic eczema in infancy, necessitating repeated courses of topical corticosteroids. Neurologically, she was diagnosed with mild autism spectrum disorder and learning disability, findings consistent with reported neurological involvement in PGM3 deficiency. 

**Table 2:** Clinical and laboratory features of PGM3 deficiency compared with the present case Clinical and laboratory features summarized from a comprehensive review of 44 cases of PGM3 deficiency reported in the literature, stratified by phenotype and frequency of manifestations. Data were derived from Yang et al. (2024) [8]. The final column presents a comparative analysis of the clinical and laboratory findings of our patient.

Category	Feature	Number of patients	Percentage		Our patient
Clinical: reported in all 44 patients					
	Recurrent infections	41	93%		Yes
	Atopy	37	84%		Yes
	Skeletal defects	27	61%		No
	Neurological disorders	21	48%		Yes
	Developmental delay	27	61%		Yes
	Failure to thrive	18	40%		No
Laboratory					
Hematological features reported in 38 out of 44 patients	Lymphopenia	25	65%		Yes
	Eosinophilia	21	54%		No
	Neutropenia	16	43%		Yes
	Anemia	13	34%		No
	Bone marrow failure	4	11%		No
	Thrombocytopenia	1	3%		No
Immunoglobulins reported in 32 out of 44 patients	Elevated serum IgE	25		78%	Yes
		Median: 9320 IU/mL (650-141,300 IU/mL)			
Lymphocyte subpopulations reported in 34 out of 44 patients	Low CD4+ T cells	32		94%	Yes
		Median: 312/µL, 94%; range: 4-913/µL			

Notably, the primary clinical indication for genetic testing in our patient was chronic neutropenia. We highlight this aspect given that chronic neutropenia is not the most typically predominant feature of the syndrome. In most reported cases, recurrent infections and atopic manifestations have been the main triggers for immunological investigation. Nonetheless, neutropenia is a relatively common finding in PGM3 deficiency, reported in approximately 43% of cases (16/38), and is observed across both HIES-like and CID/SCID-like phenotypes [4,10]. While it is not typically associated with severe sepsis, it likely contributes to the broad infectious susceptibility observed in these patients [5,7,11].

PGM3 deficiency presents with a broad phenotypic spectrum, ranging from SCID-like immunodeficiency to milder HIES-like syndromes. Given this variable presentation, a one-size-fits-all approach is not appropriate. Treatment should be tailored to the individual patient's manifestations, ranging from symptomatic management to more aggressive interventions such as hematopoietic stem cell transplantation (HSCT) in the most severe forms of the disease.

Supportive care remains the cornerstone of management, including antibiotic prophylaxis, eczema control, and treatment of infections or autoinflammatory conditions as they arise. However, HSCT has a role in selected cases, particularly in children with SCID-like presentations, where it has led to successful immunological reconstitution in some patients [5,7]. Our patient has had a favorable clinical course, experiencing few infections and requiring no frequent antibiotic therapy. Consequently, she has not needed prophylactic antibiotics or immunoglobulin replacement therapy to date.

The NGS-targeted panel that was performed on our patient included 477 genes associated with primary immunodeficiency. The analysis revealed two clinically relevant variants in the PGM3 gene. The first variant is the heterozygous missense c.1475C>T, p.(Thr492Ile), located in the phosphate-binding C-terminal protein domain. Of note, using the canonical transcript NM_015599.3 for the PGM3 gene, this missense variant is also described as c.1391C>T, p.(Thr464Ile). The c.1475C>T variant is absent from population databases (gnomAD) and has been previously reported in two unrelated individuals with PGM3 deficiency, presenting with consistent phenotypes [1,12]. Although in silico predictions are not fully concordant regarding its effect on protein function, the variant is rare, affects a conserved functional domain, and is found in trans with a loss-of-function allele in a patient with compatible clinical features. These findings support its classification as likely pathogenic. 

The second is a heterozygous deletion of approximately 167 kb located in the long arm of chromosome 6, extending across three genes: PGM3, DOP1A, and UBE3D. UBE3D and DOP1A are not currently linked to any phenotype in OMIM. The deletion encompasses nearly the entire PGM3 gene, potentially sparing only the first exon. However, the extensive loss of coding sequence likely results in a complete loss of function and absence of expression from this allele. Based on these findings, and in accordance with the American College of Medical Genetics and Genomics (ACMG), this variant is classified as likely pathogenic. 

Although variability exists, similar variants often result in comparable clinical phenotypes, suggesting a correlation between genotype and disease severity [10]. Previous studies have demonstrated that variants associated with SCID phenotypes are linked to near-absent protein expression and enzymatic function (less than 10% of wild-type levels) [7,8,10]. In contrast, variants typically associated with HIES-like presentations retain partial enzymatic activity (20-30% of wild-type levels) [7,11].

In our patient, one allele carried a large deletion encompassing nearly the entire PGM3 gene, while the second harbors a missense variant located in the C-terminal domain. Even though a functional analysis of our patient was not performed, her attenuated phenotype suggests that the missense variant may retain partial activity, potentially allowing for higher residual enzyme function. This case reinforces the emerging genotype-phenotype correlation in PGM3 deficiency, where the nature and location of variants significantly influence disease severity. These findings support the growing evidence that residual PGM3 activity is a key determinant of clinical severity in this disorder.

The genetic diagnosis of our patient was fundamental not only in clarifying the underlying cause of her clinical presentation but also in guiding long-term management and surveillance strategies. PGM3 pathogenic variants have recently been associated with an increased risk of developing myelodysplastic syndrome, highlighting the need for careful hematological monitoring over time [13]. 

Furthermore, establishing a molecular diagnosis enables accurate genetic counseling for the patient and her family, particularly in the context of family planning and risk assessment for future offspring. 

## Conclusions

PGM3 deficiency presents with a broad and variable clinical spectrum, making the diagnosis particularly challenging. This case underscores the importance of considering genetic testing in patients with atypical or non-specific features, such as chronic neutropenia, to uncover underlying inborn errors of immunity.

Genetic confirmation not only facilitates accurate diagnosis and personalized management but also plays a crucial role in guiding long-term surveillance for potential complications and enabling informed reproductive decisions. Early diagnosis is essential for tailored care, reinforcing the role of genomic analysis in evaluating patients with complex immune and hematological manifestations.

## Appendices

The next-generation sequencing (NGS)-targeted multigene panel for primary immunodeficiencies included the following genes:

ACD, ACPS, ACTB, ADA, ADA2, ADAM17, ADAR, AICDA, AIRE, AK2, ALG1, ALG12, ALPI, AP1S3, AP3B1, AP3D1, APOL1, ARHGEF1, ARPC1B, ATAD3A, ATG4A, ATM, ATP6AP1, B2M, BACH2, BCL10, BCL11B, BLM, BLNK, BLOC156, BRCA1, BRCA2, BRIP1, BTK, C1QA, C1QB, C1QC, C1R, C1S, C2, C2orf69, C3, C5, C6, C7, C8A C8B, C8G, C9, CARD11, CARD14, CARD9, CARMIL2, CASP10, CASP8, CCBE1, CD19, CD247, CD27, CD28, CD3D, CD3E, CD3G, CD4, CD40, CO40LG, CD46, CD55, CD59, CD70, CD79A, CD79B, CD81, CDC42, CDCA7, CEBPE, CFB, CFD, CFH, CFHR1, CFHR2, CFHR3, CFHR4, CFHR5, CFl, CFP, CFTR, CHD7, CHUK, CIB1, CIITA, CLCN7, CLEC7A, CLPB, COPA, COPB1, COPG1, CORO1A, CR2, CRACR2A, CREBBP, CSF?RA, CSF2RB, CSF3R, CTC1, CTLA4, CTNNBL1, CTPS1, CTSC, CXCR2, CXCR4, CYBA, CYBB, CYBC1, DBF4, DBR1, DCLRE1B, DCLRE1C, DEF6, DIAPH1, DKC1, DNAJC?1, DNASE1L3, DNASE2, DNMT38, DOCK11, DOCK2, DOCK8, DSG1, DUOX2, EFL1, ELANE, ELF4, EP300, EPGS, ERBIN, ERCC4, EXTL3, FAAP24, FADD, FANCA, FANCB, FANCC, FANCD2, FANCE, FANCF, FANCG, FANCI, FANCL, FANCM, FAS, FASLG, FAT4, FBLIM1, FCGR3A, FCHO1, IFNAR2, IFNG, IFNGR1, IFNGR2, IGHM, IGLL1, IKBKB, IKZF1, IKZF2, IKZF3, IL10, IL1ORA, IL1ORB, IL1 2B), IL12RB1, IL12RB2, IL17F, IL17RĄ, IL17RC, IL18BP, IL1RN, IL21, FCN3, FERMT1, FERMT3, FNIP1, FOXN1, FOXP3, FPR1, G6PC3, G6PD, GATA2, GFI1, GINS1, HAVCR2, HAX1, HELLS, HMOX1, HYOU1, ICOS, ICOSLG, IFIH1, IFNAR1, IL21R, IL23R, IL2RA, IL2RB, IL2RG, IL36RN, IL6, IL6R, IL6ST, IL7R, INOB0, IRAK1, IRAK4, IRF2BP2, IRF3, IRF4, IRF 7, IRF8, IRF9, I5G15, ITCH, ITG82, ITK, ITPKB, IVD, IVNS1ABP, JAGN1, JAK1, JAK3, KDM6A, KMT2A, KMT2D, KRAS, LAMTOR2, LAT, LCK, LCP2, LIG1, LIG4, LPIN2, LRBA, LSM11, LYST, MAD2L2, MAGT1, MALT1, MAN2B2, MAP1LC382, MAP3K14, MAPK8, MASP2, MBL2, MCM10, MCM4, MECOM, MEFV, MMUT, MOGS, MPEG1, MRE11, MRTFA, MS4A1, MSH6, MSN, MTHFD1, MVK, MYD88, MYSM1, NBAS, NBN, NCF2, NCF4, NCKAP1L, NCSTN, NFAT5, NFE2L2, NFKB1, NFKB2, NFKBIA, NGF, NMEJ1, NHP2, NLRC3, NLRC4, NLRP1, NLRP12, NLRP3, NLRP7, NOD2, NOP10, NOS2, NRAS, NSMCE3, NUP214, OAS1, ORAI1, OSTM1, OTULIN, PALB2, PARN, PAX1, PCCA, PCCB, PDCD1, PEPD, PGM3, PIK3CD, PIK3CG, PIK3R1, PLCG2, PLEKHM1, PMM2, PM52, PNP, POLA1, POLD1, POLD2, POLE, POLE2, POLR3A, POLR3C, POLR3F, POMP, POU2AF1, POU6F1, PRF1, PRKCD, PRKDC, PSEN1, PSENEN, PSMB4, PSMB8, P5MB9, PSMG2, PSTPIP1, PTEN, PTPN22, PTPRC, RAB27A, RAC2, RAD51, RAD51C, RAG1, RAG2, RANBP2, RASGRP1, RBCK1, REL, RELA, RELB, RFWD3, RFXS, RFXANK, RFXAP, RHOG, RHOH, RIPK1, RNASEH2A, RNASEH2B, RNASEH?C, RNF168, RNF31, RORC, RPSA, RTEL1, SAMD9, SAMD9L, SAMHD1, SASH3, SBDS, SEC61A1, SEMA3E, SERPING1, SH2D1A, SH3BP2, SH3KBP1, SIK3, SKIC2, SKIC3, SLC26A3, SlC29A3, SLC35C1, StC39A4, SLC39A7, SLCA6A1, SLC7A7, SLC9A3, SLX4, SMARCAL1, SMARCO2, SNX10, SOCS1, SP110, SPI1, SPINK5, SPPL2A, SRP54, SRP72, STAT1, STAT2, STAT3, STATSB, STAT6, STIM1, STING1, STK4, STN1, STX11, STXBP2, SYK, TAFAZZIN, TAP1, TAP2, TAPBP, TBK1, T&X1, TBX21, TCF3, TCIRG1, TCN2, TERT, TET2, TERC, TGFB1, TGFBR1, TGFBR2, THBD, TICAM1, TINF2, TRAP, TLR3, TLR7, TLR8, TMC6, TMC8, TNFAIP3, TNFRSF11A, TNFRSF13B, INFRSF13C, TNFRSF1A, TNFRSF4, TNFRSF9, TNFSF11, TNFSF12, TNFSF13, TOP2B, TP53, TPP1, TPP2, TRAC, TRAF3,  TRAF3IP2, TREX1, TRIM22, TRNTI, TTC7A, TYK2, UBA1, UBE2T, UNC119, UNC13D, UNC93B1, UNG, USB1, USP18, VPS13B, VPS45, WAS, WDR1, WIPF1, WRAP53, KIAP, XRCC2, ZAP70, Z8TB24, ZNF341, and ZNFX1.
